# NKD2 is correlated with the occurrence, progression and prognosis of thyroid carcinoma

**DOI:** 10.1186/s40001-022-00853-2

**Published:** 2022-11-08

**Authors:** Yu Gao, Yiwei Wang, Rende Guo

**Affiliations:** Department of General Surgery, Tianjin First Center Hospital, No. 94 Fukang Road, Nankai District, Tianjin, 300192 China

**Keywords:** Thyroid carcinoma, NKD2, Prognosis, Progression, RT-PCR

## Abstract

**Background:**

Thyroid carcinoma (THCA) is the most prevalent type of tumor in endocrine system. NKD2 has been increasingly evidenced to play crucial roles in many cancers, except for THCA. We herein aimed to explore the potential role of NKD2 in THCA.

**Methods:**

Totally 502 THCA patient data were downloaded from TCGA (The Cancer Genome Atlas) database. Overall survival was estimated by Kaplan–Meier method. Gene set enrichment analysis was conducted to obtain significant functional pathways. Wilcoxon rank sum test was used to determine the NKD2 expression differences among various groups. The NKD2 expression was validated in cell lines and tissue microarray.

**Results:**

Significantly higher NKD2 expression was observed in THCA samples compared with adjacent samples, which were successfully verified in cell lines and tissue microarray. Moreover, NKD2 expression gradually elevated along with the increase of TNM Stage, and NKD2 expression was significantly higher in elder THCA patients compared with young patients. NKD2 highly expressed THCA patients had worse prognosis compared with NKD2 low-expressed patients. Furthermore, 53 pathways were significantly activated in the high NKD2 expression patients compared with low NKD2 expression THCA patients.

**Conclusions:**

In summary, high NKD2 expression was probably related to the progression and poor prognosis of THCA. NKD2 is a promising prognostic biomarker and pathogenic target of THCA.

**Supplementary Information:**

The online version contains supplementary material available at 10.1186/s40001-022-00853-2.

## Background

Thyroid carcinoma (THCA) is the most prevalent type of tumor in endocrine system, accounting for approximately 4% of the newly occurred tumors [1, 2]. Over the past decades, growing THCA incidence rate has been reported in China and many other countries [3–5], which has caught our attention. Moreover, the region, race, and gender usually result in differential THCA incidence [6–8]. Generally, according to histopathology and clinical characteristics, there are four subtypes of THCA, including papillary, follicular, medullary, and undifferentiated THCA [9]. Currently, most THCA patients undergo conventional treatments like surgery, thyroid hormone replacement, or radioiodine therapy [10], but these methods are still less effective for patients with some specific THCA subtypes [11]. Furthermore, regarding early detection and diagnosis of THCA, the limitation of core needle biopsy is lack of widely recognized diagnostic criteria now [12], leading to a limited diagnostic accuracy or delayed diagnosis of THCA. Not only that, most poor prognosis or death of THCA patients result from the recurrence of tumor, metastasis, or radioiodine resistance [10]. Accordingly, it is imperative to find novel sensitively diagnostic and prognostic biomarkers for THCA.

Naked cuticle homolog 2 (NKD2) belongs to the NKD family, and it is located on chromosome 5p15.3 [13], encoding a 451-amino acid protein [14]. NKD2 or NKD2 protein mainly works by acting as a suppressor of WNT signaling pathway [15, 16]. Aberrant expression and epigenetic alterations of NKD2 have been increasingly evidenced to play crucial roles in many cancers. For example, it has been demonstrated that in many solid tumors, aberrant NKD2 expression resulted from promoter hypermethylation [17–19]. Besides, NKD2 has been frequently methylated in human breast cancer and gastric cancer [20]. In addition, it has been documented that NKD2 played important roles in the development of several tumors, like glioma, lung cancer, osteosarcoma, and gastric cancer [21]. Decreased NKD2 expression has been reported to be associated with the poor outcome in cytogenetically normal acute myeloid leukemia patients [22]. NKD2 has been indicated to be a potential prognostic biomarker and therapeutic target in hepatocellular carcinoma [15]. NKD2 could suppress the cell growth and tumor metastasis of osteosarcoma [23]. However, as far as we know, NKD2 has not been systematically explored in THCA. Therefore, it would provide more reference information for NKD2 and THCA research if we could preliminarily explore the role of NKD2 in THCA.

In this work, through comprehensive bioinformatics analyses of the THCA-related data obtained from The Cancer Genome Atlas (TCGA) database and further experimental validation, we aimed to study the possible roles of NKD2 in the occurrence, progression, or prognosis of THCA patients, in order to provide more optional choices for THCA clinical treatment strategies.

## Materials and methods

### Patient data

We have downloaded the THCA patient data from The Cancer Genome Atlas (TCGA) (https://tcga-data.nci.nih.gov/tcga/) database. Totally 502 THCA patients’ mRNA profiles and clinical data were downloaded, which included 510 THCA tissues and 58 paired adjacent tissues. Among which, one patient’s data were excluded as his/her survival information was incomplete. The the other 501 patients’ survival information was recorded completely and their data were used for further analyses. Taking the median of NKD2 expression level as the cut-off value, all THCA samples were divided into two groups, high-NKD2 expression group and low-NKD2 expression group. The clinical information of the 501 THCA patients is summarized in Table 1.

**Table1:** Clinicopathological characteristics of THCA patients from TCGA database

Characteristics	Patients(501)	
	No.	%
Age		
≤ 46 (median)	252	50.30%
> 46 (median)	249	49.70%
Pathologic stage		
i	281	56.09%
ii	52	10.38%
iii	111	22.16%
iii	111	22.16%
Unknown	2	0.40%
Survival time		
Long(> 5 years)	95	18.96%
Short(< 5 years)	406	81.04%
OS status		
Dead	16	3.19%
Alive	485	96.81%

### Cell lines and culture

Totally three cell lines were used in our study, including normal human thyroid follicular epithelial cell line NTHY-ORI3-1 (purchased from BeNa Culture Collection, Beijing, China), THCA cell line SW579 (purchased from Cell Resource Center, Beijing, China), and human anaplastic thyroid carcinoma 8305C (purchased from National Collection of Authenticated Cell Cultures, Shanghai, China). Then, NTHY-ORI3-1, SW579, and 8305C were cultured in RPMI-1640 medium (GIBCO, 31870082), L-15 medium (GIBCO, 41300039), and Eagle’s minimal essential medium (EMEM) complete medium (Invitrogen, 11090081), respectively. All cell lines were cultured in 90% medium and 10% FBS at 37 °C with an atmosphere of 5% CO_2_.

### Gene set enrichment analysis (GSEA)

GSEA analysis was conducted in GSEA software (version: #4.0), on the basis of c2.cp.kegg.v7.0.symbols in Molecular Signatures Database (MSigDB). The P value was adjusted according to Benjamini and Hochberg (BH) method, and the pathways with adjust P value < 0.05 were taken as significantly enriched KEGG pathways.

### qRT-PCR

The total RNA was extracted with TRIzol (Tiangen, DP431, Beijing, China), which was measured with a spectrophotometer (Thermo, New York, USA). The reagents used in qPCR experiments: reverse transcription kit (Tiangen Biochemical, KR118, Beijing, China) and SYBR detection reagent (Tiangen, FP205, Beijing, China). The following thermocycling conditions were applied for qPCR: denaturation at 95˚C for 15 min; 40 cycles of 95 °C for 10 s, 60 °C for 20 s, 72 °C for 20 s. The internal reference was β-actin. The primer sequences are listed in Table 2. There were three repeats per sample. The relative mRNA expression was calculated based on formula 2^−△△CT^.

**Table 2:** Primer sequences for RT-PCR

Genes	Forward primer (5'-3')	Reverse primer (5'-3')	Product length (bp)	Tm(℃) (°C)
NKD2	GAGGACCAGTGTCCCCTACAG	CTCCGTCATCTGCGCTGAG	91	61
β-actin	CCTGGCACCCAGCACAAT	GGGCCGGACTCGTCATAC	144	60

### Western blot analysis

The methods we used in western blot analysis were consistent with the previous common methods [24]. The reagents we used were shown as below: BCA protein concentration detection kit (Solarbio, cat#PC0020, Beijing, China), microplate reader (SpectraMax, M5, USA), primary antibody NaKed 2 polycIonal AnTibody (Abcam, ab153717, 1:300, UK), internal reference GAPDH (Bioss, cat#bs-2188R, 1:1000, Beijing, China), and secondary antibody Goat anti-rabbit IgG-HRP (Bioss, cat#bs-0295G-HRP, 1:4000, Beijing, China). Software Gelpro32 was used for gray value analyses.

### Tissue microarray and immunohistochemical analyses

The THCA tissue microarray (HThyP120CS02, OUTDO Biotech, Shanghai, China) contained 4 normal thyroid tissue samples, and 116 cases of papillary thyroid carcinoma and the paired paracancerous tissues. The clinical information of the tissue microarray is shown in Additional file 1: Table S1 (all detailed information could be available at https://www.superchip.com.cn/biology/tissue.html). The sections were scanned using Pannoramic 250FLASH (3DHISTECH, Hungary) and captured with Aipathwell (Servicebio, China), then the integral optic density (IOD) was calculated. The primary antibody NaKed 2 polycIonal AnTibody (ab33654A36, 1:30, Abcam, UK), the secondary antibody Goat Anti-rabbit IgG/HRP (SE134, Solarbio, 1:150, Beijing, China), and histochemistry kit DAB (DAKO, K5007, USA) were used in our experiment.

### Statistical analyses

The NKD2 expression differences between THCA samples, adjacent samples, and samples with various clinicopathological characteristics were determined by Wilcoxon rank sum test. The multivariate Cox regression analysis was done to determine the independent prognostic indicators for THCA. All statistical analyses were conducted using R software v3.5.2. *P* value < 0.05 was considered as statistically significant difference.

## Results

### Higher expression of NKD2 in THCA patients

**Fig. 1:**
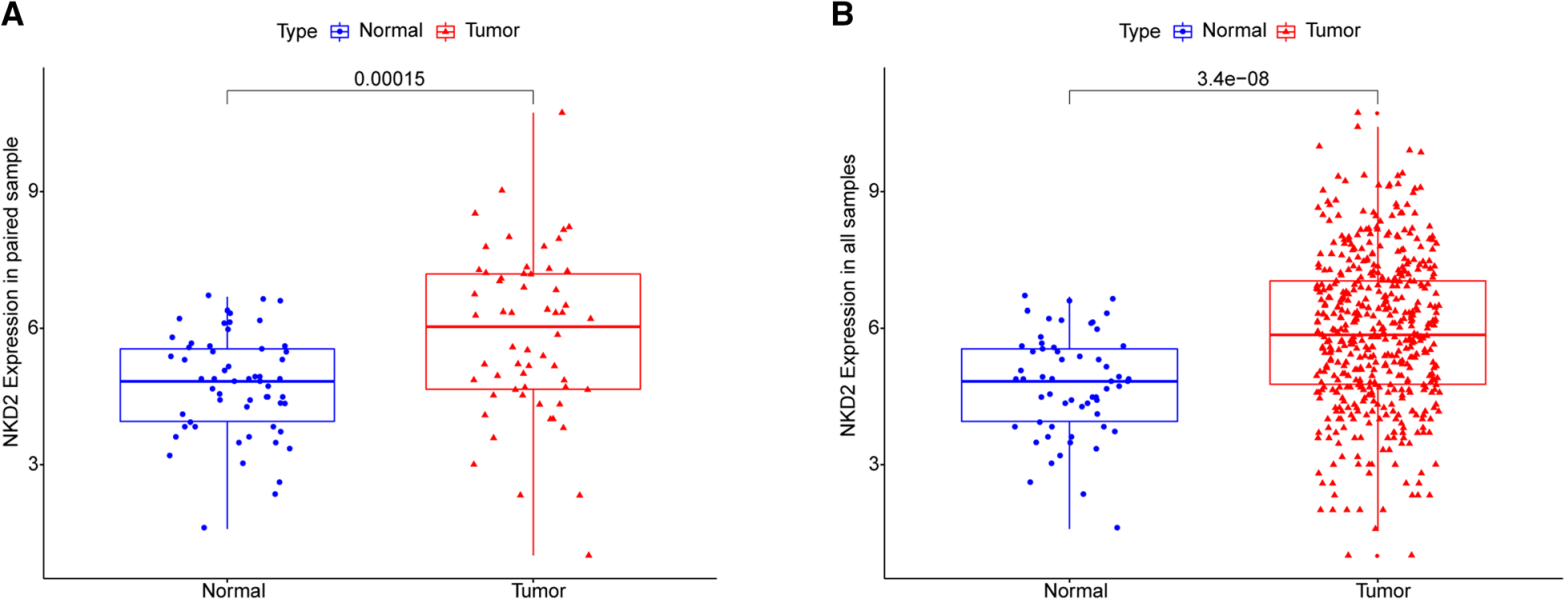
The NKD2 expression levels in THCA samples and adjacent samples. **A** The NKD2 expression levels in paired THCA and adjacent samples. **B** NKD2 expression levels in all THCA and adjacent samples

Firstly, the expression of NKD2 in THCA samples and adjacent samples were compared to explore the potential correlation between NKD2 expression and THCA onset. The results showed that compared with in adjacent samples, the expression of NKD2 in paired THCA samples (*N* = 58) was significantly higher (*P* = 0.00015) (Fig. 1A). Moreover, significantly higher NKD2 expression was also observed in all THCA samples (N = 510) compared with adjacent samples (*N* = 58) (*P* = 3.4e–08) (Fig. 1B). Our results indicated that high expression of NKD2 was related to the occurrence of THCA.

### Correlation between NKD2 expression and clinicopathologic features

**Fig. 2:**
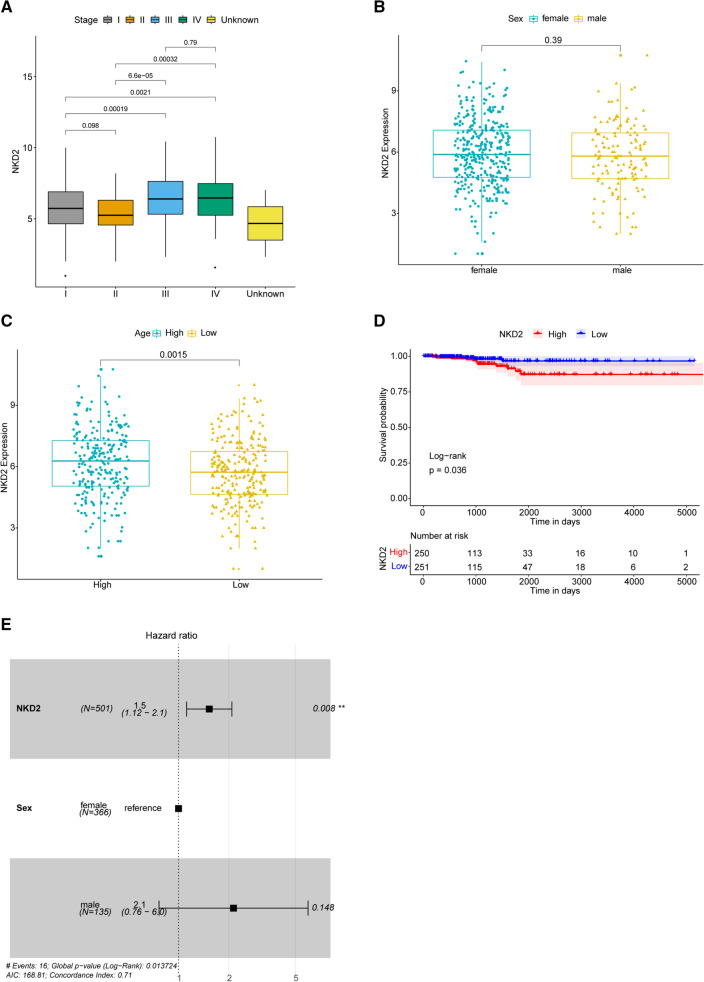
The correlation between NKD2 expression and clinicopathological characteristics, prognosis of THCA patients. **A** NKD2 expression levels in different TNM stages of THCA samples. **B** NKD2 expression levels in male and female THCA patients. **C** NKD2 expression levels in different ages. **D** Kaplan–Meier survival curves of high and low NKD2 expression THCA patients. P value was calculated based on log-rank test. X-axis: time (days); Y-axis: survival probability. **E** The results of multivariate Cox regression analysis

Subsequently, the correlation between NKD2 expression and various corresponding THCA clinicopathological characteristics was analyzed based on Wilcoxon rank sum test. We found that the expression of NKD2 gradually increased along with the increase of TNM Stage, besides, there were significant differences between Stage I vs. Stage III, Stage II vs. Stage III, Stage II vs. Stage IV, and Stage I vs. Stage IV (*P* value < 0.05, Fig. 2A). Moreover, elevated NKD2 expression was significantly correlated with Age of THCA patients. NKD2 expression was significantly higher in elder THCA patients compared with young patients (*P* value < 0.05, Fig. 2C). However, there was no significant correlation between NKD2 expression and sex (*P* value > 0.05, Fig. 2B).

### THCA patients with high NKD2 expression had poor prognosis

To further study the prognostic value of NKD2 in THCA patients, the survival analysis was conducted on high and low NKD2 expression THCA patients, based on the data in TCGA database. Comparing with patients with low NKD2 expression, patients with high NKD2 expression had a poorer overall survival (*p* = 0.036, *HR* = 0.32, 95% CI 0.12–0.85) (Fig. 2D). Additionally, a multivariate Cox regression analysis was conducted including NKD2 expression and sex, which suggested that NKD2 was still significantly associated with the prognosis of THCA patients (Fig. 2E).

### NKD2-related results of GSEA

**Fig. 3:**
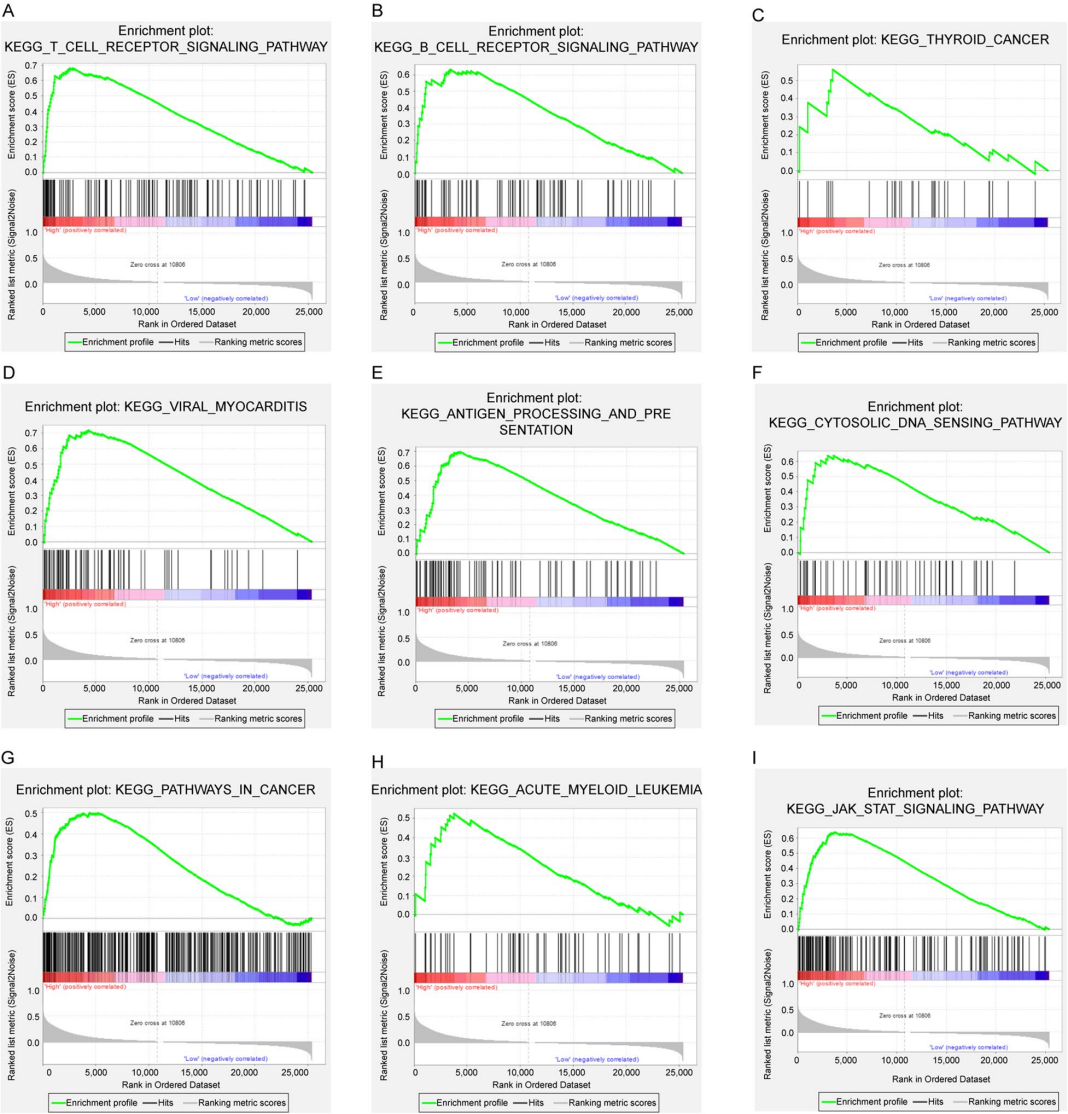
Significantly activated KEGG pathways in NKD2 high-expressed THCA patients, based on GSEA. **A **T cell receptor signaling pathway, (**B**) B cell receptor signaling pathway, (**C**) thyroid cancer, (**D**) viral myocarditis, (**E**) antigen processing and presentation, (**F**) cytosolic DNA sensing pathway, (**G**) pathways in cancer, (**H**) acute myeloid leukemia, (**I**) jak/stat signaling pathway. The top 9 significantly enriched pathways (P < 0.05)

Furthermore, signaling pathways significantly enriched in NKD2 high-expressed THCA patients were identified via GSEA enrichment analysis, compared with NKD2 low-expressed patients. The KEGG pathways with adjusted *P* value < 0.05 were screened as significantly enriched pathways. There were totally 53 pathways significantly enriched in the NKD2 high-expressed group, and the top 9 most significant pathways included T cell receptor signaling pathway, B cell receptor signaling pathway, thyroid cancer, viral myocarditis, antigen processing and presentation, cytosolic DNA sensing pathway, pathways in cancer, acute myeloid leukemia, and JAK/STAT signaling pathway (Fig. 3A–I). The results suggested that thyroid cancer pathway and several other cancer-related pathways were significantly activated in NKD2 highly expressed THCA patients. The detailed results of GSEA are shown in Additional file 2: Table S2.

### High NKD2 expression validated in THCA cell lines and tissue microarray

**Fig. 4:**
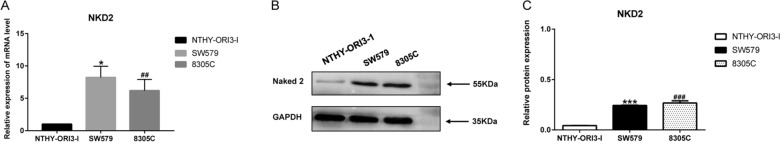
The NKD2 expression in cell lines. **A** NKD2 mRNA was upregulated in THCA cell lines (* P < 0.05, vs. SW579; ## P < 0.001, vs. 8305C). **B, C** The NKD2 protein expression levels in THCA cell lines (*** P < 0.001, vs. SW579; ### P < 0.001, vs. 8305C)

**Fig. 5:**
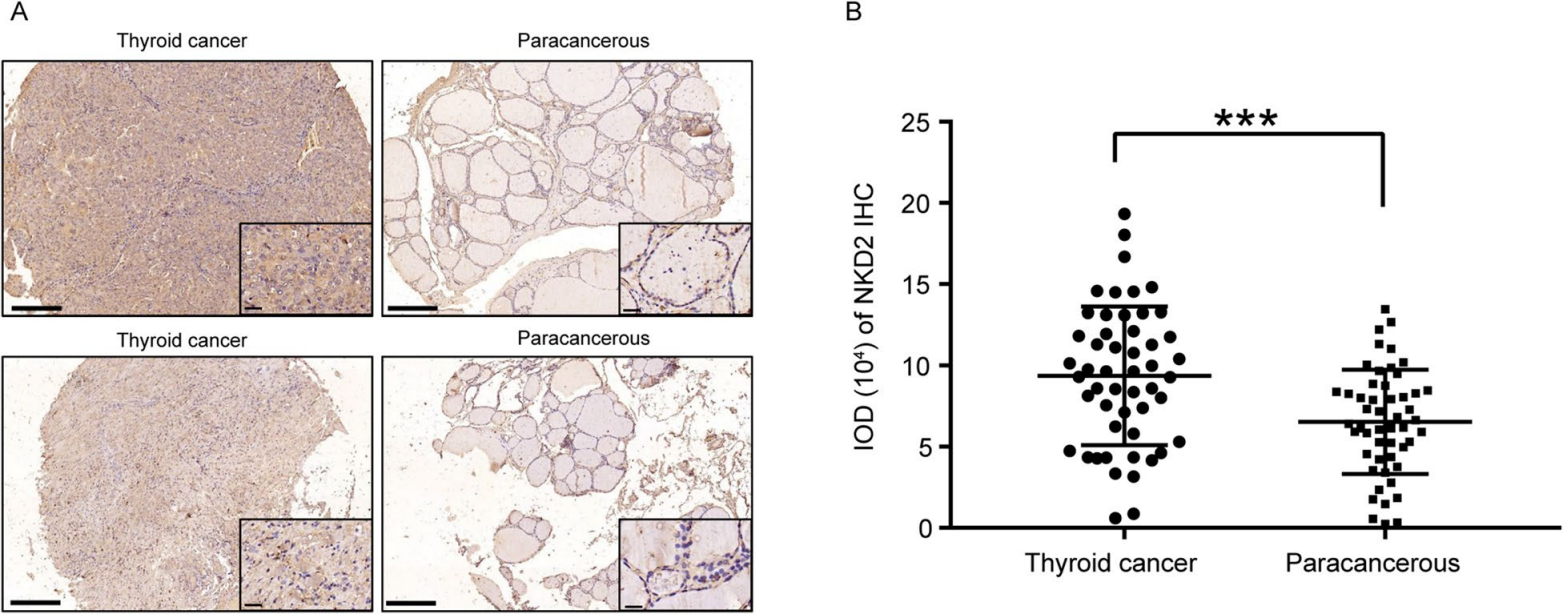
The expression of **A** NKD2 in **B** THCA tissue microarray.

Subsequently, we have further validated our results in cell lines and tissue microarray. Firstly, in three cell lines, the relative NKD2 mRNA expression was significantly higher in THCA cell lines compared with in normal cells (Fig. 4A), and the tendency of protein expression was consistent (Fig. 4B–C, Additional file 3). Moreover, the results of IHC analyses suggested that NKD2 expression was still significantly higher in THCA tissue samples compared with adjacent samples (Fig. 5A–B). Our findings have been successfully validated in cell lines and clinical samples.

## Discussion

Over the past decades, increasing incidence of THCA has been widely reported, especially in females [25]. Despite significant advances have been achieved in THCA therapy, effective treatment strategies for some specific types of THCA are still lacking. In the present study, we have explored the potential role of NKD2 in the progression and prognosis of THCA. High NKD2 expression was probably related to the progression and occurrence of THCA. Moreover, NKD2 highly expressed THCA patients had worse prognosis compared with patients with low NKD2 expression.

The role of NKD2 in many tumors has been reported [26, 27], except for THCA. Consequently, we aimed to better understand the role of NKD2 in THCA, on the basis of data in TCGA database. Firstly, we found that significantly higher NKD2 expression was observed in THCA samples (including paired THCA vs. adjacent samples and all THCA vs. adjacent samples), indicating the potential pathogenic role of NKD2 in THCA. Our results were also successfully validated in cell lines and clinical samples, and were compatible with some previous reports directly or indirectly. NKD2 has been evidenced to serve as a suppressor of WNT signaling pathway. Additionally, the role of NKD2 involving development, progression, or prognosis in several tumors has been demonstrated, like glioma, lung cancer, osteosarcoma, and gastric cancer [19, 21]. Despite we have explored the role of NKD2 in THCA for the first time, only a small part of the NKD2 function was found in our study. NKD2 was previously reported to be regulated by promoter region methylation [18, 20] and it was the target of some lncRNAs or miRNAs in the regulation of several cancers [26, 28], which should be further studied in our future research. Furthermore, elevated NKD2 expression was associated with the increase of TNM Stage, which suggested indirectly that NKD2 might contribute to the progression of THCA, as a pathogenic factor. On this point, NKD2-related underlying mechanisms in THCA deserved further investigation, and its detailed function is probably conducive to studying potential therapy strategies targeting NKD2. Moreover, there was no significant correlation between NKD2 expression and gender, although it has been widely reported that a higher incidence of THCA was observed in females than in males [29]. While NKD2 expression seems to be not correlated with gender, which might result from limited samples in this research. Additionally, THCA patients with high NKD2 expression had a worse prognosis compared with patients with low NKD2 expression. The prognostic value of NKD2 has been demonstrated in some other tumors, for example, hepatocellular carcinoma [15], ovarian cancer [30], acute myeloid leukemia [22]. More recently, Wu et al. have documented the prognostic value of several WNT signaling pathway molecules in colorectal cancer (CRC), including NKD2, and the inhibition of NKD2 could inhibit the tumor cell growth of CRC [31]. Higher NKD2 expression was significantly correlated with poorer survival of CRC [31], thereby their results partly supported our findings in THCA. Collectively, NKD2 is a poor prognostic factor for THCA, which is firstly reported in our research.

Furthermore, the GSEA analysis was conducted to better understand the potential function of NKD2 in THCA. Totally, 53 pathways were significantly activated in the NKD2 high-expressed group compared to the NKD2 low-expressed group. Notably, 9 various cancer-related pathways, including Thyroid cancer pathway, were significantly enriched, which implied that NKD2 was actually involved in cancer. Moreover, some of the enriched pathways have been recently evidenced to involve in the anticancer activities of THCA, for instance, JAK/STAT3 signaling pathway [32, 33]. Not only that, several known pathways that were involved in oncogenic mechanisms in thyroid cancer initiation or progression were also found to be significantly activated, including VEGF signaling pathway [34], Toll-like receptor signaling pathway [35], MAPK signaling pathway [36]. Nonetheless, the precise role of NKD2 in THCA still needs to be clarified.

Although the pathogenic role and prognostic value of NKD2 in THCA have been demonstrated in our study for the first time, there were still some limitations in our work. The THCA-related data in TCGA database are limited, and more public data will be helpful to strengthen our results. Secondly, we have preliminarily explored the role of NKD2 via in silico analysis and wet-lab validation, but deepening underlying mechanisms regarding NKD2 are not well investigated herein.

## Conclusions

In conclusion, our study explored the important role of NKD2 in THCA patients for the first time. On the basis of integrated bioinformatics analyses of THCA-related data in TCGA database, we also conducted further validation experiments in cell lines and clinical tissues to further confirm that high NKD2 expression was correlated with the progression and prognosis of THCA. Collectively, our findings indicate that NKD2 is a promising prognostic biomarker and pathogenic target of THCA.

## Supplementary Information

**Additional file 1: Table S1.** Clinical features of thyroid cancer patients.

**Additional file 2: Table S2.** GSEA result.

**Additional file 3.** The original results of western blot.

## Data Availability

The datasets generated and analyzed for this study can be found in the TCGA database (https://tcga-data.nci.nih.gov/tcga/).

## Abbreviations

THCA: Thyroid carcinoma
NKD2: Naked cuticle homolog 2
TCGA: The Cancer Genome Atlas
GSEA: Gene set enrichment analysis
