# Supplementary material for: NKD2 is correlated with the occurrence, progression and prognosis of thyroid carcinoma
Source: Eur J Med Res. 2022 Nov 8;27:235. doi: 10.1186/s40001-022-00853-2 (PMC9641892; doi:10.1186/s40001-022-00853-2)
Supplement: Supplementary file 1 — Additional file 1: Table S1. Clinical features of thyroid cancer patients. [file 40001_2022_853_MOESM1_ESM.docx]

Table S1. Clinical features of throid cancer patients

| **Sex** | **Age** | **Tumor Size (cm×cm×cm)** | **T** | **N** | **M** | **Pathological Grade** |
| --- | --- | --- | --- | --- | --- | --- |
| Male | 36 | 1×0.8×0.8 | T1a | N0 | M0 | I |
| Female | 29 | 1.5×1.5×1 | T1b | N0 | M0 | I |
| Female | 40 | 1.6×1.1×1 | T1b | N0 | M0 | I |
| Female | 38 | 1.5×1×1 | T1b | N0 | M0 | I |
| Male | 34 | 1.8×1.5×0.8 | T1b | N0 | M0 | I |
| Female | 41 | 1.7×1.5×1.5 | T1b | N0 | M0 | I |
| Female | 40 | 2×1.5×1 | T1b | N0 | M0 | I |
| Female | 41 | 2 | T1b | N0 | M0 | I |
| Male | 27 | 2 | T1b | N0 | M0 | I |
| Female | 43 | 2 | T1b | N0 | M0 | I |
| Male | 41 | 1.5×1.5×1 | T1b | N0 | M0 | I |
| Female | 20 | 2×1.5×1.5 | T1b | N0 | M0 | I |
| Male | 40 | 2×2×1.5 | T1b | N0 | M0 | I |
| Male | 40 | 2.5×1.5×1.2 | T2 | N0 | M0 | I |
| Female | 35 | 3.1×1.8×1.5 | T2 | N0 | M0 | I |
| Female | 27 | 3×3×2.5 | T2 | N0 | M0 | I |
| Male | 43 | 2.5 | T2 | N0 | M0 | I |
| Male | 38 | 1.8×1.8×1.7 | T1b | N1 | M0 | I |
| Female | 36 | 1.5×1.2×1 | T1b | N1 | M0 | I |
| Female | 42 | 2×1.5×1 | T1b | N1 | M0 | I |
| Male | 40 | 3×2×2 | T2 | N1 | M0 | I |
| Female | 34 | 2.5×2×1.5 | T2 | N1 | M0 | I |
| Female | 31 | 2.5×2.5×1.5 | T2 | N1 | M0 | I |
| Male | 14 | 1.5×1×0.5 ;5×4×2.5 | T3 | N1 | M0 | I |
| Female | 53 | ①1×1×0.8 ;②0.3×0.3×0.2 ;0.2×0.2×0.1 | T1a | N0 | M0 | I |
| Male | 48 | 1×1×0.8 | T1a | N0 | M0 | I |
| Male | 52 | ①0.8×0.7×0.5 ;②2×1.5×1.5 | T1b | N0 | M0 | I |
| Female | 51 | ①1.7×1.2×1 ;②0.3×0.3×0.3 | T1b | N0 | M0 | I |
| Female | 54 | 1.7×1.5×1.2 | T1b | N0 | M0 | I |
| Male | 63 | 2×1.5×1.5 | T1b | N0 | M0 | I |
| Female | 56 | 1.5×1.5×1 | T1b | N0 | M0 | I |
| Female | 70 | 2.5×1.5×1 | T2 | N0 | M0 | II |
| Female | 57 | 3×2×2 | T2 | N0 | M0 | II |
| Female | 91 | 3.5×3.5×3 | T2 | N0 | M0 | II |
| Female | 82 | 2.5 | T2 | N0 | M0 | II |
| Male | 60 | 2.5 | T2 | N0 | M0 | II |
| Female | 50 | 2.5×2×2 | T2 | N0 | M0 | II |
| Female | 55 | 1.3×0.7×0.7 | T1b | N1a | M0 | III |
| Female | 50 | 2.5×2×1.7 ;0.2×0.2×0.2 | T2 | N1a | M0 | III |
| Female | 48 | 0.6 | T3 | N0 | M0 | III |
| Female | 68 | 1.5×1×1 | T1b | N1b | M0 | IV |
| Male | 47 | 2.3×2×2 | T2 | N1b | M0 | IV |
| Male | 45 | 1.5×1×1 ;0.3×0.3×0.2 | T4 | N1b | M0 | IV |
| Female | 52 | 2×2×1.5 | T4 | —— | M0 | IV |
| Male | 62 | 1.9×1.7×1.7 | T1b | N1 | M0 | III－IV |
| Female | 47 | 1.5×1.4×1.4 | T1b | N1 | M0 | III－IV |
| Male | 50 | 1.8×1.5×1 | T1b | N1 | M0 | III－IV |
| Female | 51 | 1.5×1.4×1.3 | T1b | N1 | M0 | III－IV |
| Female | 57 | 2×2×1 | T1b | N1 | M0 | III－IV |
| Female | 79 | 3.5×0.5 | T2 | N1 | M0 | III－IV |
| Male | 62 | ①3.5×3.5×2.2 ;②1.2×1.2×1 ;1.2×1.2×0.8 ;0.3×0.3×0.3 | T2 | N1 | M0 | III－IV |
| Male | 47 | 0.5-3 | T2 | N1 | M0 | III－IV |
| Male | 59 | 2.5×2×2 | T2 | N1 | M0 | III－IV |
| Female | 48 | 2.5×2.2×1.7 | T2 | N1 | M0 | III－IV |
| Female | 58 | 3 | T2 | N1 | M0 | III－IV |
| Male | 59 | 3.5×2.5×2 | T2 | N1 | M0 | III－IV |
| Male | 57 | ①0.5×0.5×0.3 ;②2.5×2×2 | T2 | N1 | M0 | III－IV |
| Female | 50 | 5×4×2 | T3 | N1 | M0 | III－IV |
