# Supplementary material for: NKD2 is correlated with the occurrence, progression and prognosis of thyroid carcinoma
Source: Eur J Med Res. 2022 Nov 8;27:235. doi: 10.1186/s40001-022-00853-2 (PMC9641892; doi:10.1186/s40001-022-00853-2)
Supplement: Supplementary file 2 — Additional file 2: Table S2. GSEA result. [file 40001_2022_853_MOESM2_ESM.docx]

Table S2. GSEA result

| NAME | NES | FDR q-val |
| --- | --- | --- |
| KEGG_T_CELL_RECEPTOR_SIGNALING_PATHWAY | 1.9679703 | 0.011186934 |
| KEGG_B_CELL_RECEPTOR_SIGNALING_PATHWAY | 1.9687262 | 0.012305628 |
| KEGG_THYROID_CANCER | 1.9485633 | 0.012329184 |
| KEGG_VIRAL_MYOCARDITIS | 1.9096992 | 0.012988648 |
| KEGG_ANTIGEN_PROCESSING_AND_PRESENTATION | 1.9140121 | 0.013140841 |
| KEGG_CYTOSOLIC_DNA_SENSING_PATHWAY | 1.9214497 | 0.013221422 |
| KEGG_PATHWAYS_IN_CANCER | 1.8948677 | 0.013399627 |
| KEGG_ACUTE_MYELOID_LEUKEMIA | 1.8753215 | 0.013537006 |
| KEGG_JAK_STAT_SIGNALING_PATHWAY | 1.9690794 | 0.01367292 |
| KEGG_MELANOMA | 1.8702184 | 0.013819198 |
| KEGG_REGULATION_OF_ACTIN_CYTOSKELETON | 1.8852228 | 0.013924948 |
| KEGG_TOLL_LIKE_RECEPTOR_SIGNALING_PATHWAY | 1.9730792 | 0.013941464 |
| KEGG_NATURAL_KILLER_CELL_MEDIATED_CYTOTOXICITY | 1.8982568 | 0.013972604 |
| KEGG_FOCAL_ADHESION | 1.9846122 | 0.01413481 |
| KEGG_CELL_ADHESION_MOLECULES_CAMS | 1.8761733 | 0.014249481 |
| KEGG_APOPTOSIS | 1.8519711 | 0.014250504 |
| KEGG_BLADDER_CANCER | 1.8469603 | 0.014311257 |
| KEGG_FC_EPSILON_RI_SIGNALING_PATHWAY | 1.8538568 | 0.01445137 |
| KEGG_FC_GAMMA_R_MEDIATED_PHAGOCYTOSIS | 1.827706 | 0.015062673 |
| KEGG_EPITHELIAL_CELL_SIGNALING_IN_HELICOBACTER_PYLORI_INFECTION | 1.8328484 | 0.015399291 |
| KEGG_P53_SIGNALING_PATHWAY | 1.8289669 | 0.015501312 |
| KEGG_COMPLEMENT_AND_COAGULATION_CASCADES | 1.8160152 | 0.015641205 |
| KEGG_MAPK_SIGNALING_PATHWAY | 1.8358859 | 0.015729984 |
| KEGG_ENDOCYTOSIS | 1.8168267 | 0.01605978 |
| KEGG_GLIOMA | 1.9850134 | 0.016490612 |
| KEGG_VEGF_SIGNALING_PATHWAY | 1.8082213 | 0.016948847 |
| KEGG_NOD_LIKE_RECEPTOR_SIGNALING_PATHWAY | 1.9980444 | 0.018506942 |
| KEGG_SYSTEMIC_LUPUS_ERYTHEMATOSUS | 1.7877082 | 0.020078 |
| KEGG_AUTOIMMUNE_THYROID_DISEASE | 1.7741663 | 0.022324922 |
| KEGG_LEUKOCYTE_TRANSENDOTHELIAL_MIGRATION | 2.0017166 | 0.023133678 |
| KEGG_CHEMOKINE_SIGNALING_PATHWAY | 2.0173671 | 0.023225814 |
| KEGG_HEMATOPOIETIC_CELL_LINEAGE | 1.7503996 | 0.025810858 |
| KEGG_PATHOGENIC_ESCHERICHIA_COLI_INFECTION | 1.7310413 | 0.029528983 |
| KEGG_PRION_DISEASES | 2.0336232 | 0.030109525 |
| KEGG_INTESTINAL_IMMUNE_NETWORK_FOR_IGA_PRODUCTION | 1.7312804 | 0.030294549 |
| KEGG_TYPE_I_DIABETES_MELLITUS | 1.7168776 | 0.031916395 |
| KEGG_CHRONIC_MYELOID_LEUKEMIA | 1.7173792 | 0.032547187 |
| KEGG_GLYCOSPHINGOLIPID_BIOSYNTHESIS_GANGLIO_SERIES | 1.704451 | 0.03268027 |
| KEGG_HYPERTROPHIC_CARDIOMYOPATHY_HCM | 1.7047552 | 0.033379454 |
| KEGG_ECM_RECEPTOR_INTERACTION | 1.7056044 | 0.034020223 |
| KEGG_WNT_SIGNALING_PATHWAY | 2.067703 | 0.03435898 |
| KEGG_NON_SMALL_CELL_LUNG_CANCER | 1.6948732 | 0.034590226 |
| KEGG_GLYCOSAMINOGLYCAN_DEGRADATION | 1.695668 | 0.034925435 |
| KEGG_TGF_BETA_SIGNALING_PATHWAY | 1.6905918 | 0.0351595 |
| KEGG_SMALL_CELL_LUNG_CANCER | 1.685816 | 0.035191026 |
| KEGG_ASTHMA | 1.6858925 | 0.035878006 |
| KEGG_LYSOSOME | 1.6743429 | 0.038381774 |
| KEGG_MELANOGENESIS | 1.6668857 | 0.03920388 |
| KEGG_PROSTATE_CANCER | 1.6691301 | 0.039378446 |
| KEGG_PROTEASOME | 1.6586006 | 0.041423593 |
| KEGG_RIG_I_LIKE_RECEPTOR_SIGNALING_PATHWAY | 1.6521294 | 0.04170561 |
| KEGG_CELL_CYCLE | 1.6540781 | 0.04188579 |
| KEGG_RENIN_ANGIOTENSIN_SYSTEM | 1.6296556 | 0.049647715 |
