# Supplementary figures and images for: NKD2 is correlated with the occurrence, progression and prognosis of thyroid carcinoma
Source: Eur J Med Res. 2022 Nov 8;27:235. doi: 10.1186/s40001-022-00853-2 (PMC9641892; doi:10.1186/s40001-022-00853-2)

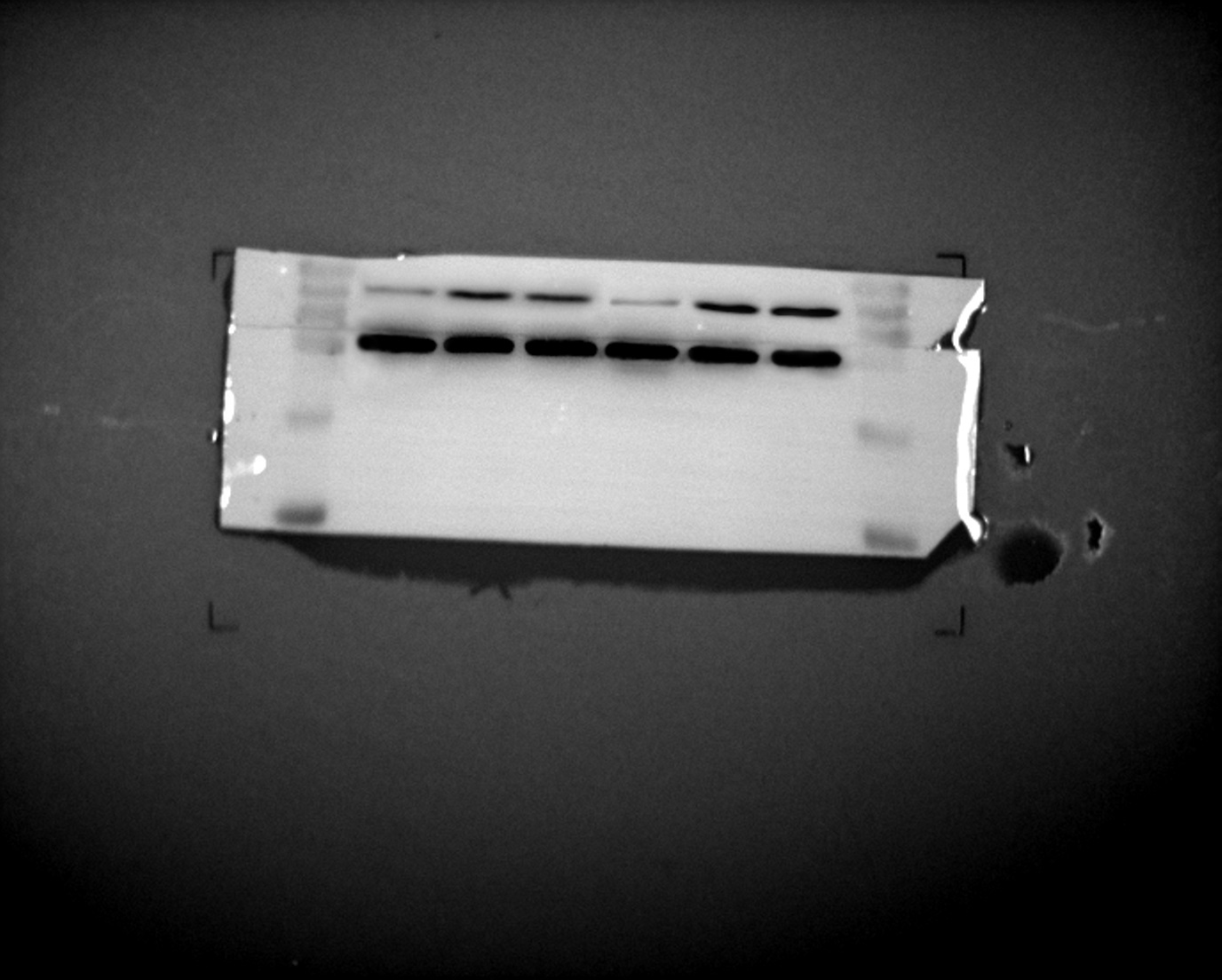


**Naked2**

**GAPDH**

**8305C**

**SW579**

**SW579**

**8305C**

**NTHY-ORI3-1**

**NTHY-ORI3-1**

Supplement: Supplementary file 3 — Additional file 3. The original results of western blot. [file 40001_2022_853_MOESM3_ESM.docx]
